# DDAH1 Deficiency Attenuates Endothelial Cell Cycle Progression and Angiogenesis

**DOI:** 10.1371/journal.pone.0079444

**Published:** 2013-11-18

**Authors:** Ping Zhang, Xin Xu, Xinli Hu, Huan Wang, John Fassett, Yuqing Huo, Yingjie Chen, Robert J. Bache

**Affiliations:** 1 Cardiovascular Division, Department of Medicine, University of Minnesota Medical School, Minneapolis, Minnesota, United States of America; 2 Institute of Molecular Medicine, Peking University, Beijing, China; 3 Vascular Biology Center, Department of Cellular Biology and Anatomy, Medical College of Georgia, Georgia Health Sciences University, Augusta, Georgia, United States of America; Sun Yat-sen University Medical School, China

## Abstract

Asymmetric dimethylarginine (ADMA) is an endogenous inhibitor of nitric oxide (NO) synthase (NOS). ADMA is eliminated largely by the action of dimethylarginine dimethylaminohydrolase1 (DDAH1). Decreased DDAH activity is found in several pathological conditions and is associated with increased risk of vascular disease. Overexpression of DDAH1 has been shown to augment endothelial proliferation and angiogenesis. To better understand the mechanism by which DDAH1 influences endothelial proliferation, this study examined the effect of DDAH1 deficiency on cell cycle progression and the expression of some cell cycle master regulatory proteins. DDAH1 KO decreased in vivo Matrigel angiogenesis and depressed endothelial repair in a mouse model of carotid artery wire injury. DDAH1 deficiency decreased VEGF expression in HUVEC and increased NF1 expression in both HUVEC and DDAH1 KO mice. The expression of active Ras could overcome the decreased VEGF expression caused by the DDAH1 depletion. The addition of VEGF and knockdown NF1 could both restore proliferation in cells with DDAH1 depletion. Flow cytometry analysis revealed that DDAH1 sRNAi knockdown in HUVEC caused G1 and G2/M arrest that was associated with decreased expression of CDC2, CDC25C, cyclin D1 and cyclin E. MEF cells from DDAH1 KO mice also demonstrated G2/M arrest that was associated with decreased cyclin D1 expression and Akt activity. Our findings indicate that DDAH1 exerts effects on cyclin D1 and cyclin E expression through multiple mechanisms, including VEGF, the NO/cGMP/PKG pathway, the Ras/PI3K/Akt pathway, and NF1 expression. Loss of DDAH1 effects on these pathways results in impaired endothelial cell proliferation and decreased angiogenesis. The findings provide background information that may be useful in the development of therapeutic strategies to manipulate DDAH1 expression in cardiovascular diseases or tumor angiogenesis.

## Introduction

Asymmetric dimethylarginine (ADMA) is an endogenous inhibitor of nitric oxide (NO) synthase (NOS). Increased plasma levels of ADMA are associated with endothelial dysfunction in patients with vascular disease or risk factors [1]. ADMA is eliminated by the action of dimethylarginine dimethylaminohydrolase (DDAH). There are two isoforms of DDAH (DDAH1 and DDAH2). DDAH1 is the major enzyme to degrade ADMA [2]. Thus, DDAH2 levels were unchanged in DDAH1 knock out (KO) mice, but these mice lacked DDAH activity, had increased plasma and tissue ADMA levels, decreased NO synthesis and increased blood pressure [2].

Nitric oxide is known to inhibit apoptosis and increase proliferation and migration of endothelial cells [3]. NO stimulates endothelial cell DNA synthesis and proliferation via cGMP-dependent transcription and is a central regulator of angiogenesis. We have found that the endogenous NOS inhibitor, ADMA, inhibits endothelial cell proliferation and angiogenesis [4], [5]. Cell proliferation is determined by cell cycle progression and is tightly controlled by master regulatory proteins including cyclins, cyclin dependent kinases (CDKs), their substrate proteins, the Cdk inhibitors (CKI), and the tumor-suppressors p53 and pRb [6]. These families comprise the basic regulatory machinery responsible for catalyzing cell cycle transition and checkpoint control.

The importance of the DDAH/ADMA pathway on angiogenesis has been demonstrated in a number of systems. DDAH1 transgenic mice show increased blood vessel formation in a mouse model of hind limb ischemia as well as in the fibrovascular disc system [7], [8], and demonstrate augmented endothelial regeneration after femoral artery injury [9]. Conversely, aortic ring explants from DDAH1 global KO and endothelial specific KO mice exhibit reduced endothelial sprouting in Matrigel [4], [10].

DDAH1 is known to regulate endothelial cell proliferation by degrading ADMA, thereby augmenting NO production and activating the cGMP/PKG pathway. Thus, DDAH1 knockdown increased ADMA levels, depressed NO production, and decreased cell proliferation; conversely overexpression of DDAH1 decreased ADMA, increased NO production, and increased cell proliferation. In addition to these effects on the NO/cGMP/PKG pathway, we have found that DDAH1 also acts to activate Akt in an NO/cGMP independent manner [4].

DDAH activity is decreased in several pathological conditions. Thus, in diabetic rats aortic DDAH activity was significantly reduced and negatively associated with plasma ADMA levels [11]. Myocardial DDAH activity is decreased in the setting of congestive heart failure [12]. A loss-of-function polymorphism of DDAH1 has been found in humans that is associated with increased risk of thrombotic stroke and coronary heart disease [13]. The present study was performed to examine the mechanism by which DDAH1 influences endothelial cell proliferation and the vascular response to injury. Based on our prior data, we hypothesized that the absence of DDAH1 would alter cell cycle progression, impair endothelial cell proliferation and angiogenesis, and result in an impaired response to vascular injury.

## Methods

### Experimental Animals

DDAH1 global KO and endothelial cell specific KO mice were generated in our lab [2], [10]. All animal experiments were approved by the University of Minnesota Animal Care and Use Committee in accordance with the Association for Assessment and Accreditation of Laboratory Animal Care (AAALAC) guidelines. All surgery was performed under anesthesia using an intraperitoneal injection of ketamine (80 mg/kg body weight) and xylazine (5 mg/kg) (Phoenix Scientific, Inc., St. Joseph, MO).

### In vivo Matrigel Angiogenesis

Mice (8 weeks) were anesthetized. Matrigel (BD Bioscience, GFR BD Matrigel matrix, phenol red –free) 300 µl supplemented with heparin (64 unit/ml) was injected subcutaneously into wild type (WT) control mice and DDAH1 global KO mice using a 25G needle [14]. Each animal was injected at 3 sites. After 2 weeks, the animals were sacrificed and the Matrigel plugs were removed. The angiogenic response was evaluated by measurement of the hemoglobin content in the Matrigel plugs. Hemoglobin was mechanically extracted from the Matrigel plugs in water and measured using the Drabkin method by spectrophotometric analysis (Sigma, Chemical Co., St Louis, Mo.). For histological study, the Matrigel plug was fixed with formalin overnight, embedded in paraffin, sectioned and stained with Hematoxylin and Eosin (H&E) using the staining kit from ScyTek Laboratories. Each group consisted of four animals and the experiments were repeated 3 times.

### Mouse Carotid Artery Wire Injury Model

The arterial wire injury was performed as described [15]. Briefly, mice (8 weeks of age) were anesthetized. After midline neck incision, the left external carotid artery was tied off distally and a 0.014-inch flexible angioplasty guide wire was advanced 1 cm into the common carotid artery via a transverse arteriotomy. Endothelial denudation was achieved by five passes of the guide wire with a rotating motion.

### Evans Blue Staining of Injured Arteries

Endothelial regeneration of the injured artery was evaluated by staining of the denuded areas with Evans blue dye (Sigma). 5 days after carotid injury, mice were anesthetized and then injected intravenously with 300 µl of saline containing 2% Evans blue dye. Ten minutes later, mice were euthanized, followed by perfusion of 4% paraformaldehyde/PBS for 5 min. The injured artery was opened longitudinally and placed en face on Parafilm. The Evans blue-stained luminal area indicated the area not covered with endothelial cells. The endothelial regenerated area was calculated as the percentage of the non-blue area over the total injured luminal surface of the artery.

### Cell Culture and Transfection

Primary human umbilical artery endothelial cells (HUVEC) (Lonza) were maintained in medium EGM2–MV (Lonza) under 5% CO_2_ in a humidified incubator at 37°C. Cells at passage 4 to 7 were used for experiments. Cells with 30% confluence were transfected with Lipofectamin 2000 (LF-2000, Invitrogen) according to the protocol provided by the manufacturer. After 5 hours incubation, the growth medium was replaced.

Mouse embryonic fibroblast (MEF) cells were isolated from 13.5-day-old embryos from WT control and DDAH1 global KO mice. Briefly, the head and internal viscera were removed, and the remaining embryonic tissues were minced and digested by incubation in 0.25% trypsin –EDTA solution (Invitrogen). After incubation for 45 min at 37°C with gentle agitation, trypsin was inactivated by adding 3 volumes of complete medium (DMEM supplemented with 10% fetal bovine serum, 100 U/ml penicillin, and 100 µg/ml streptomycin). Isolated cells were harvested and cultured at 37°C in a humidified atmosphere of 95% air/5% CO2. MEFs from the 2 to 4 passages were used in all experiments.

### Western Blot

Tissue homogenate and cell lysate in lysis buffer were resolved on 8–12% SDS-polyacrylamide gels, followed by routine western blot procedures. The following primary antibodies were used: cyclin A, cyclin B, cyclin D, cyclin E, CDC2, CDC25C, p27Kip1, p21, VEGF, NF1 (Santa Cruz), β-actin (Sigma), Ras, P–Akt-473 and total Akt (Cell Signaling). DDAH1 antibody (mouse) was a gift from Dr. Kimoto [10].

### Cell Proliferation Assay

Cell proliferation was determined by MTT assay as previously described [4].

### Fluorescence-activated Cell Sorting (FACS) Analysis

HUVEC were seeded (1.5×10^5^ cells/35 mm dish) and cultured overnight before sRNAi transfection. After transfection cells were cultured for 24 hours; at that time BrdU (10 μ M) was added and culture continued for an additional 16 hours. Cells were fixed and labeled with APC-conjugated anti-BrdU and 7-ADD using the APC BrdU Flow kit (BD Pharmingen) and analyzed using a FACScalibur cytometer. Later, the MEF cell cycle was analyzed using propidium iodine (Sigma) stain.

### Statistical Analysis

Results are presented as mean ± standard deviation. Significance testing between groups was performed using Student’s unpaired t-test. P<0.05 was considered to be statistically significant.

## Results

### 1. Decreased Angiogenesis in DDAH1 Global KO Mice using the *in vivo* Matrigel Angiogenesis Assay

**Figure 1:**
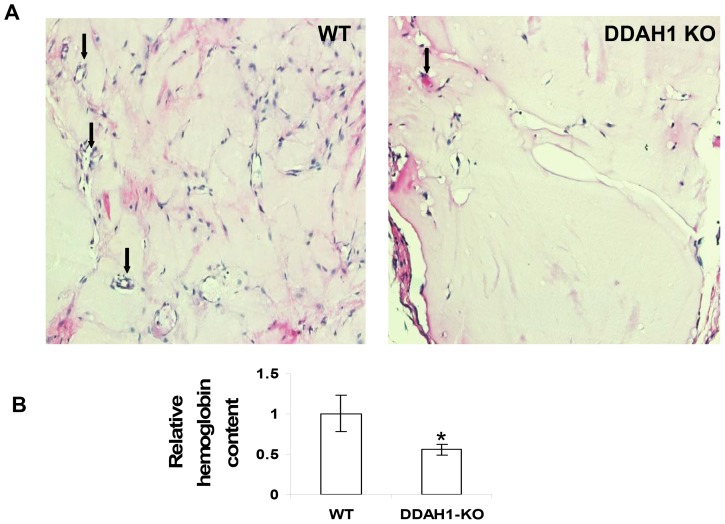
Decreased angiogenesis in DDAH1 KO mice. (A) H&E staining of Matrigel plugs. Representative images show fewer microvessels in the KO mice (20x magnification). Arrows show microvessels. (B) The angiogenic response was evaluated by measurement of hemoglobin content in the Matrigel plugs after two weeks. Each group consisted of four animals and the experiments were repeated 3 times (*p<0.05).

The DDAH1 global KO mice generated in our lab are viable but have increased plasma and tissue ADMA levels, decreased NO synthesis, and increased systemic blood pressure [2]. We previously reported that endothelial sprouting was decreased in cultured *ex vivo* aortic rings from DDAH1 global KO mice [4]. In the current study, we assessed *in vivo* vessel growth by measuring the hemoglobin content and H&E staining of Matrigel plugs 2 weeks after subcutaneous implantation. We found fewer microvessels (Figure 1A) and lower hemoglobin content in Matrigel plugs from the DDAH1 KO mice than in the WT mice (Figure 1B), consistent decreased angiogenesis.

### 2. Impaired Endothelial Repair after Vessel Injury

**Figure 2:**
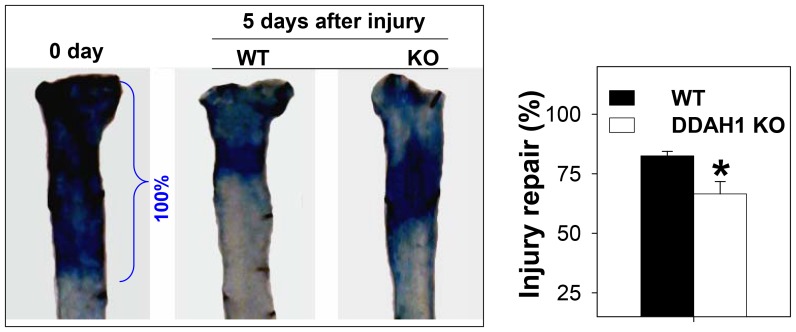
DDAH1 deficiency decreased endothelial regeneration. Evans blue dye staining of injured carotid arteries shows areas that were not covered with newly generated endothelial cells. Each group consisted of 6 animals (*p<0.05).

Konishi [9] reported that endothelial repair after vascular injury was enhanced in transgenic mice overexpressing DDAH1. Using the mouse carotid artery wire injury model, we studied endothelial regeneration after denudation. In comparison with WT mice, we found that endothelial regeneration was impaired in DDAH1 endothelial specific KO mice (KO vs. WT: 66.7±4.95% vs. 83.3±1.08% of injured surface area, n = 6, p<0.05) (Figure 2).

### 3. Effects of DDAH1 Knockdown on Cell Cycle Distribution

**Figure 3:**
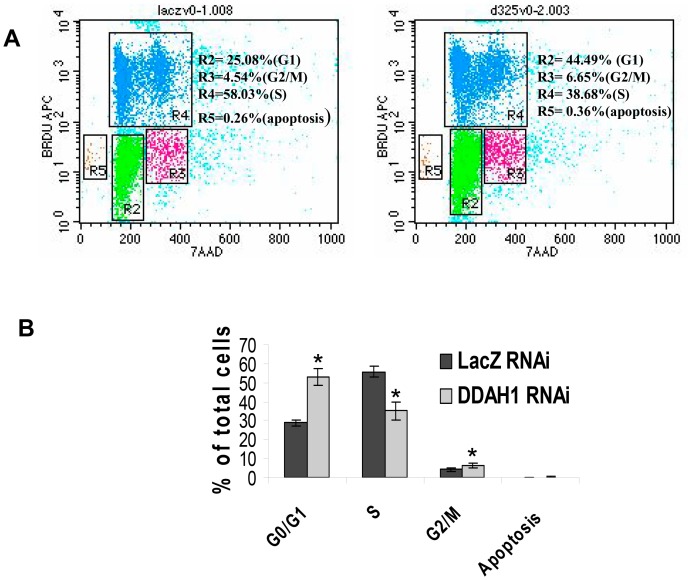
DDAH1 deficiency caused HUVEC arrest in G1 and G2/M phases of the cell cycle. HUVEC were transfected with control sRNAi and DDAH1 sRNAi. 48 hours later the cells were fixed and labeled with APC-conjugated anti-BrdU and 7-ADD using the APC BrdU flow kit and measured by FACS. (A) Representative histograms showing cell cycle distribution of HUVEC. (B) A significant accumulation in G1 phase and G2/M phase was observed in HUVEC with DDAH1 sRNAi. (n = 4, *p<0.05).

We have found that DDAH1 regulates endothelial cell proliferation both by degrading ADMA, thereby increasing NO production, and by activating Akt in a NO-cGMP independent manner [4]. Since both NO and Akt have potential effects on cell cycle progression [16], [17], we studied the cell cycle distribution in DDAH1 knockdown cells using flow cytometry analysis. As reported before, knockdown of DDAH1 with sRNAi transfection inhibited HUVEC proliferation [4]. In control cells, the distribution of the cell population in the G1 phase was 27.23±3.08%, G2/M phase 4.88±0.6% and S phase 53.38±3.46% (Figure 3). In DDAH1 sRNAi transfected cells, G1 phase was increased to 33.22±1.53% and G2/M phase to 6.95±0.59%, while cells in S phase were decreased to 43.96±3.27% (p<0.02, n = 4). These data provide evidence of G1 and G2/M arrest after DDAH1 knockdown.

### 4. Genes Related to Cell Cycle Progression

**Figure 4:**
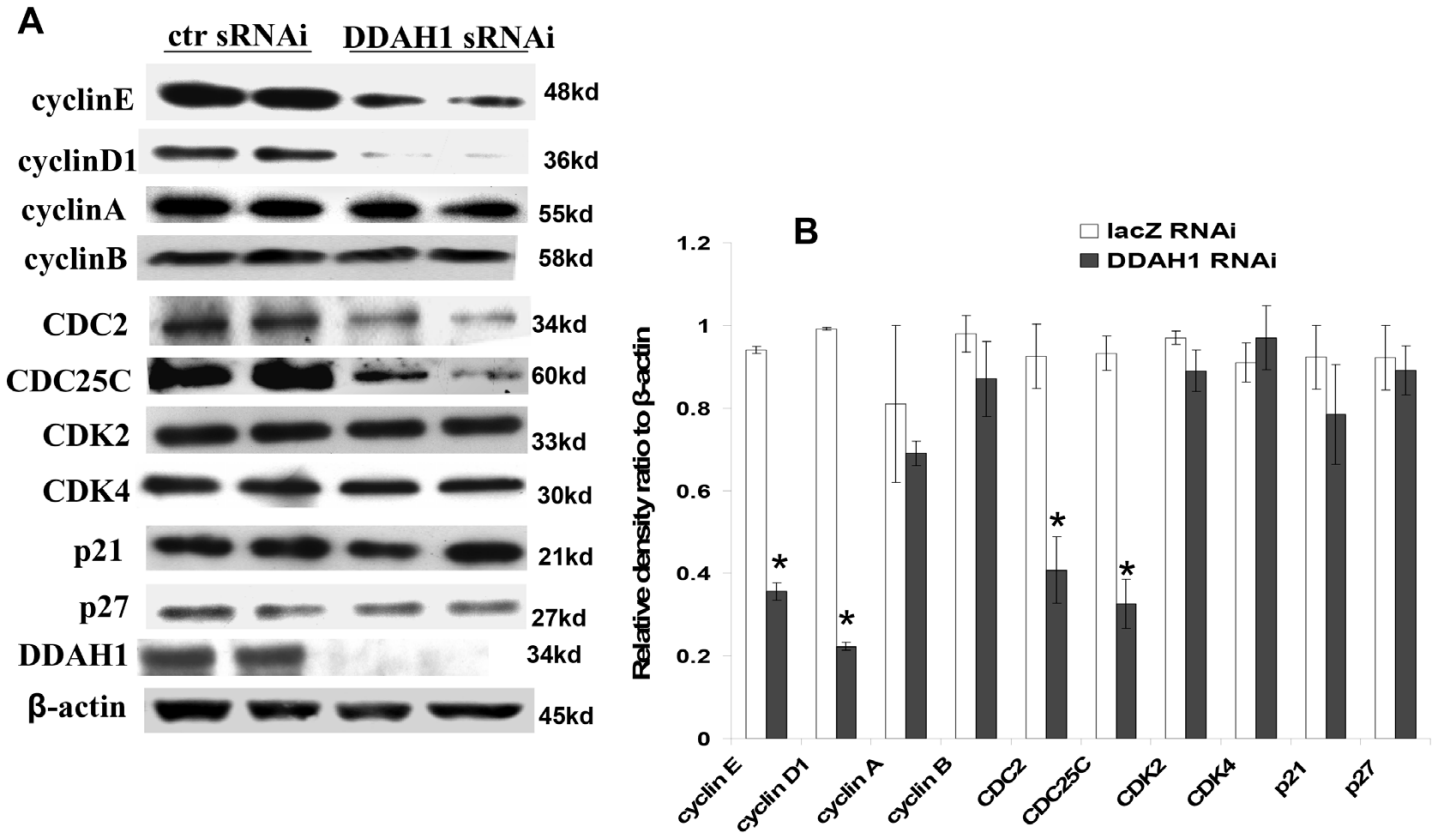
The effect of DDAH1 deficiency on the expression of cell cycle regulatory proteins. (A) HUVEC were lysed 48 h after transfection with sRNAi and the lysates were subjected to western blot analysis. Each blot is a representative of 4 similar experiments. (B) Relative protein contents (*p<0.05).

Cell cycle progression is tightly controlled by master regulatory proteins including cyclins, cyclin dependent kinases (Cdks) and Cdk inhibitors (CKI). Consequently, we investigated expression of several of these genes involved in the regulation of cell cycle progression. As shown in Figure 4A–B, DDAH1 knockdown for 48 hours resulted in decreased expression of cyclin E, cyclin D1, CDC2 and CDC25C, while the levels of cyclin A, cyclin B, CDK2, CDK4, p21cip1 and p27kip1 were unchanged. Cell cycle progression is controlled by several CDK complexes, including cyclin D1/CDK4 and cyclin E/CDK2 in the G1/S transition, and cyclin B1/CDK1 in the G2/M transition. CDC2 and CDC25C proteins play an important role in the S phase and the G2/M progression of the cell cycle. The activities of these complexes depend on the balance between cyclins and CKIs. The findings suggest that DDAH1 knockdown resulted in down regulation of CDC2, CDC25C, cyclin D1 and cyclin E which contributed to the G1 and G2/M arrest in the HUVEC.

### 5. Altered Cell Cycle Progression in MEF Cells from DDAH1 Global KO Mice

**Figure 5:**
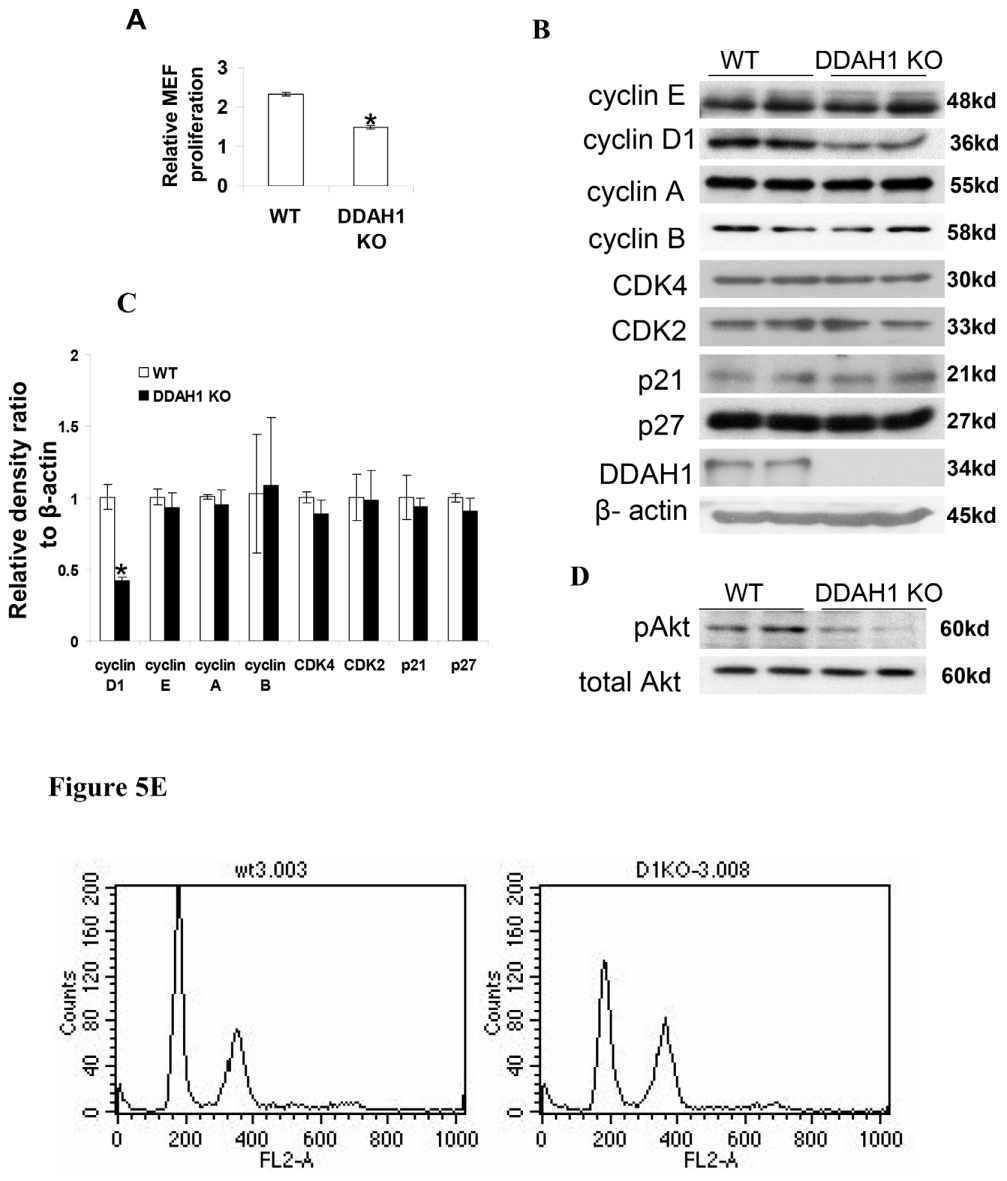
MEF cells from DDAH1 KO mice have decreased cell proliferation. (A) MTT assay shows that cell proliferation was decreased in the MEF from DDAH1 KO mice compared to WT control mice. (B) Western blot of cell cycle regulating proteins. Each blot is representative of 4 experiments. (C) Relative protein contents. Comparing MEF from DDAH1 KO mice and WT control mice, the cyclin D1 level was decreased while the levels of cyclin A, cyclin B, cyclin E, CDK2, CDK4, p21 and p27 were unchanged. (D) Western blots of pAkt-473 and total Akt. The pAkt-473 level was decreased in DDAH1 KO MEF. Each blot is representative of 4 experiments. (E) Cell cycle distribution of MEF. PI stain and FACS assay showed the accumulation of the G2/M population in MEF from DDAH1 KO mice compared to control WT mice (*p<0.05).

In order to better understand the function of DDAH1, we generated DDAH1 global KO mice in our laboratory. We isolated MEF cells from WT control and DDAH1 global KO mice for study of the role of DDAH1 in cell cycle progression. The proliferation of MEF was significantly decreased in cells obtained from DDAH1 global KO mice (Figure 5A) in comparison with cells from WT mice. Using flow cytometry analysis we found that in MEF from WT mice, 51.5±1.3% of the cells were in G1, whereas 35.7±2.3 of the cells were in G2/M. In contrast, 45.8±2.4% of the DDAH1 global KO MEF were in G1, whereas 45.5±2.6% of the cells were in G2/M, indicating that a large part of the population of the DDAH1 global KO MEF were delayed in G2/M phase (Figure 5E).

### 6. Cyclin D1 Expression is Decreased in DDAH1 Global KO MEF

When we analyzed the expression levels of some of the proteins related to cell cycle progression, we found that cyclin D1 expression was decreased in DDAH1 global KO MEF cells, whereas cyclin E, cyclin A, cyclin B, CDK2, CDK4, and P21, P27 were unchanged (Figure 5B–C). We also found that pAkt-473 level was decreased in the DDAH1 global KO MEF cells (Figure 5D), which was consistent with our previous study in endothelial cells [4].

### 7. DDAH1 Altered VEGF Expression in HUVEC

**Figure 6:**
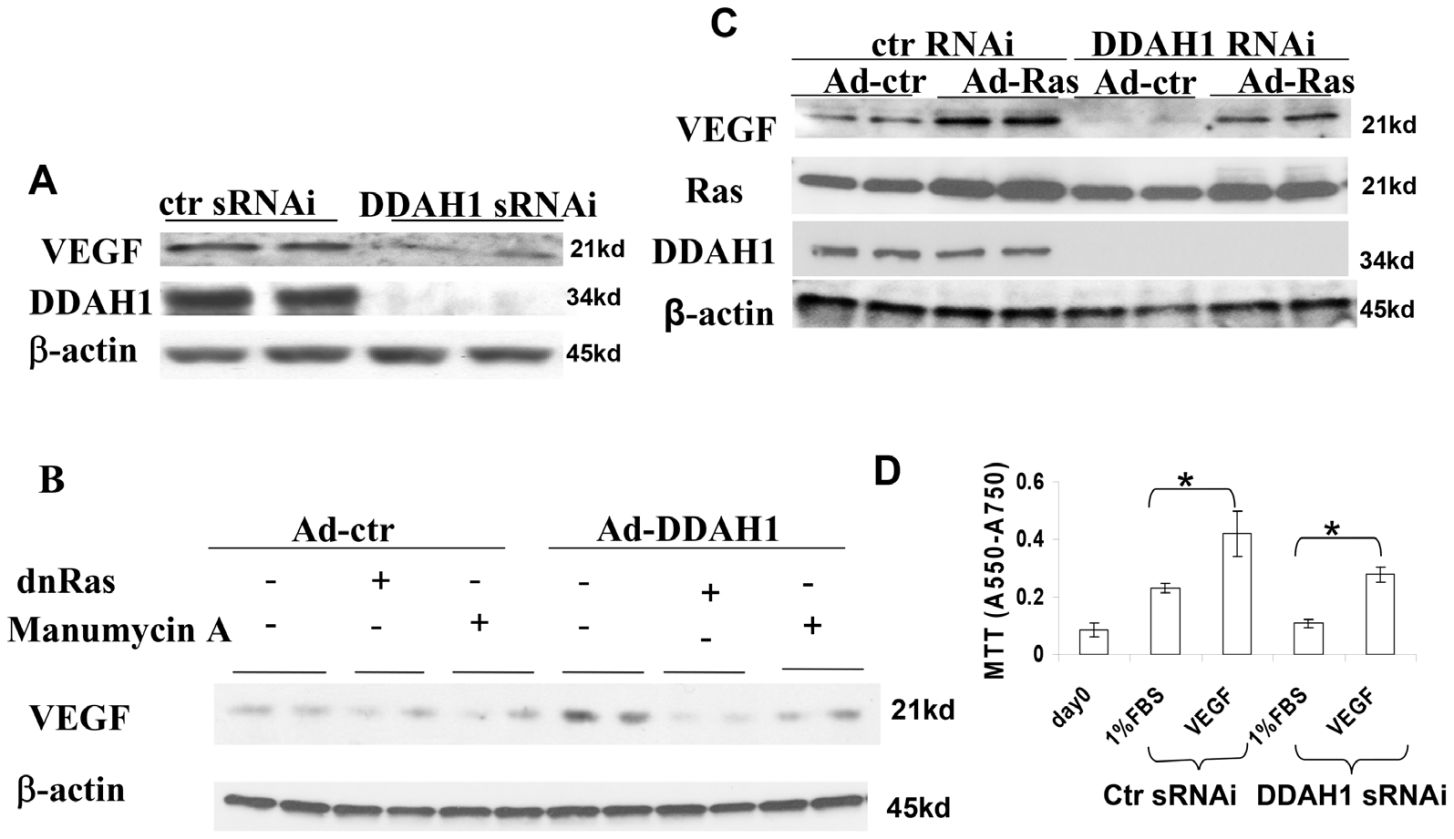
The effect of DDAH1 on VEGF expression in HUVEC. (A) DDAH1 deficiency decreased VEGF protein level in HUVEC. (B) Blocking Ras activation by Ad-dnRas and the Ras inhibitor manumycin A inhibited the up-regulation of VEGF by DDAH1. (C) Overexpression of Ras increased VEGF expression. (D) Treatment with VEGF (50 ng/ml) for 2 days increased proliferation in HUVEC (n = 4, *p<0.05).

Since vascular endothelial growth factors (VEGFs) are master regulators of angiogenesis, vascular development and blood vessel function [18], we examined the effect of DDAH1 on VEGF expression. We found VEGF expression was decreased in HUVEC with DDAH1 knockdown (Figure 6A). PI3K/Akt is known to up regulate VEGF expression [19]. We previously reported that [4] DDAH1 can activate the Ras/PI3K/Akt pathway, and that inhibition of Ras by Ad-dnRas infection or with manumycin A was able to block the Akt activation. Consequently, we studied the effect of DDAH1 overexpression on VEGF in HUVEC. We found that DDAH1 overexpression caused an increase of VEGF expression, and that Ras inhibition by Ad-dnRas infection or the inhibitor manumycin A blocked the increase in VEGF expression (Figure 6B). These results indicate that regulation of VEGF by DDAH1 is a downstream effect of Akt activation. When we overexpressed active Ras with ad-Ras G12V infection (MOI 20) in HUVEC with DDAH1 knockdown, we found that active Ras increased VEGF expression in the control cells, which is consistent with a previous study in cancer cells [20]. We also found that expression of active Ras could overcome the decreased VEGF expression caused by the DDAH1 deficiency (Figure 6C). Since VEGF is important for cell proliferation, and DDAH1 knockdown decreased VEGF expression and cell proliferation, we tested whether the addition VEGF could restore proliferation in cells with DDAH1 depletion. We found that treatment with VEGF (50 ng/ml) for 2 days significantly increased HUVEC proliferation in both control cells and in cells with DDAH1 knockdown (Figure 6D).

### 8. DDAH1 Effects on NF1 Expression

**Figure 7:**
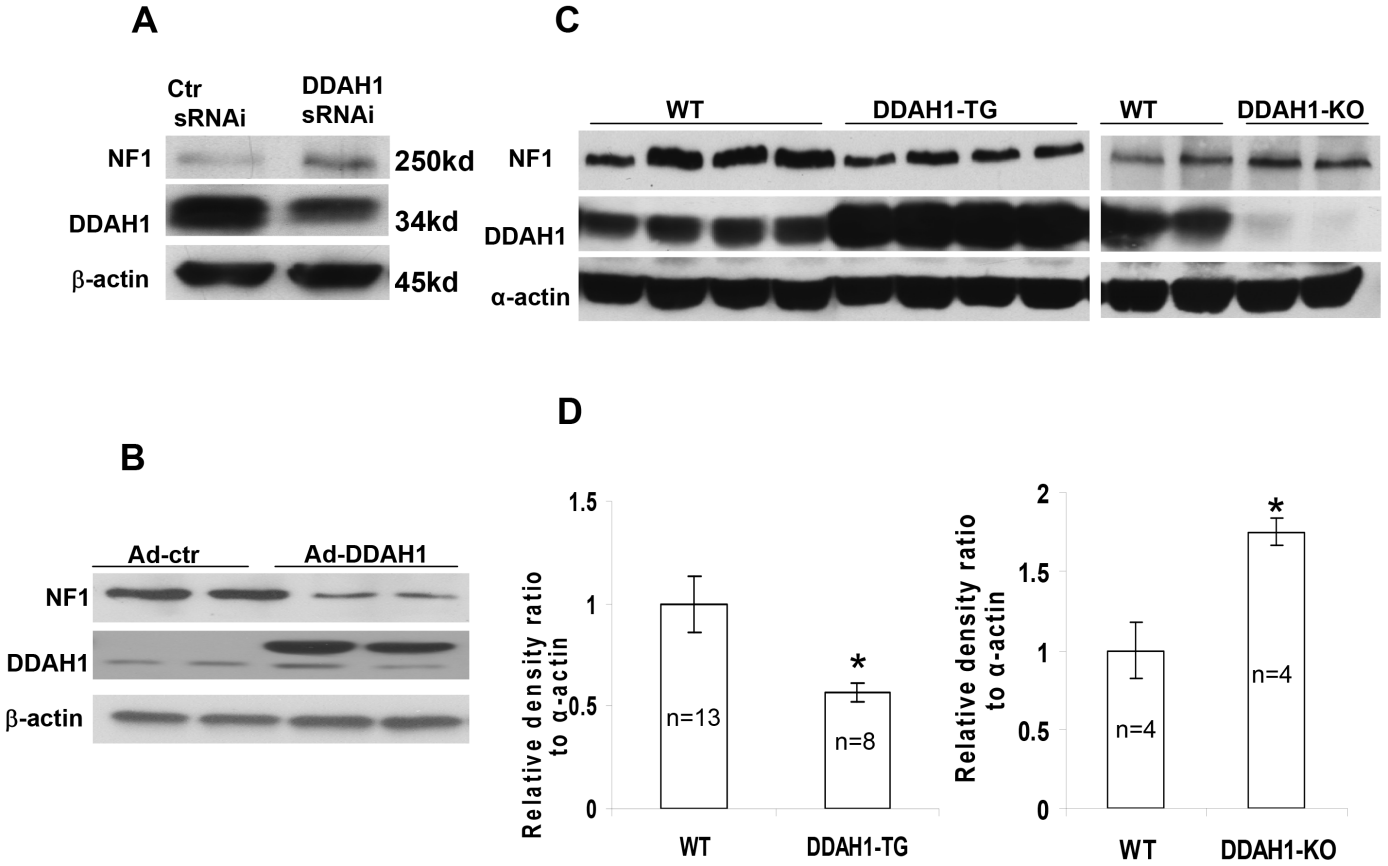
The effect of DDAH1 on NF1 expression. (A) Knockdown DDAH1 increased NF1 expression in HUVEC. (B) Overexpression of DDAH1 decreased NF1 expression in HUVEC. (C) NF1 levels were decreased in heart tissue from DDAH1 transgenic mice, but increased in DDAH1 KO mice. (n = 4–13, *p<0.05).

NF1 is a tumor suppressor which has specific-Ras GTPase activating protein activity (GAP) by which it can regulate the mTOR pathway [21]. DDAH1 is known to bind to NF1 [22]. Using the Matrigel plug assay, NF1+/− mice were found to have an increased angiogenic response to VEGF [23] and increased angiogenesis *in vivo* [24], [25]. Since the effect of DDAH1 on NF1 expression is unknown, we decided to study this effect by western blot. As shown in Figure 7A–B, DDAH1 knockdown increased the NF1 level in HUVEC, while DDAH1 overexpression decreased the NF1 level. We subsequently examined NF1 levels in heart tissue from DDAH1 transgenic mice and DDAH1 global KO mice. We found increased NF1 levels in DDAH1 global KO mice and decreased NF1 levels in DDAH1 transgenic mice (Figure 7C–D), consistent with the results in HUVEC.

### 9. Knockdown of NF1 Increases Akt Activity and Cell Proliferation in HUVEC

**Figure 8:**
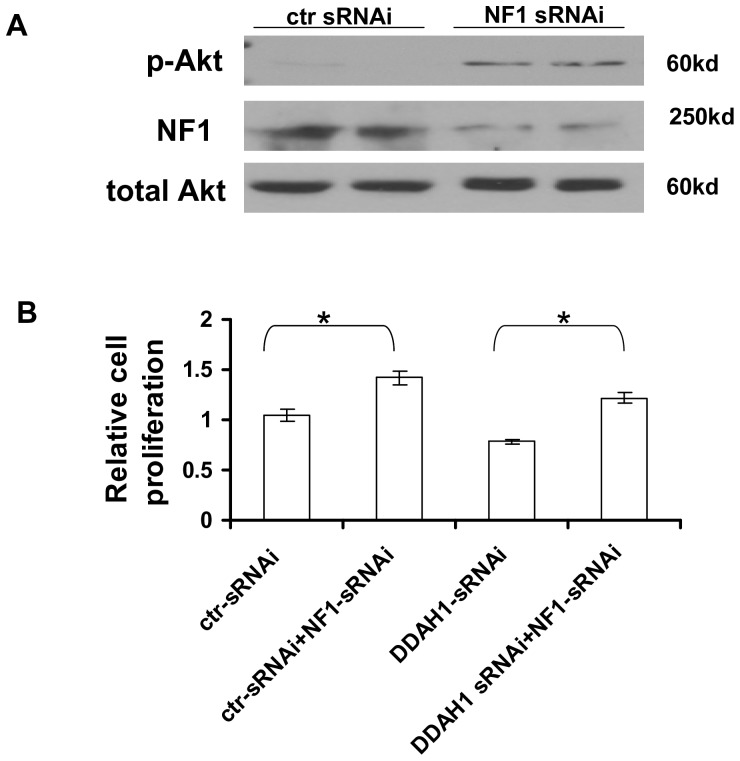
Knockdown of NF1 in HUVEC. (A) Knockdown of NF1 increased pAkt-473 in HUVEC. (B) Knockdown of NF1 increased HUVEC proliferation in both control cells and cells with DDAH1 knockdown (n = 4, *p<0.05).

We previously reported that DDAH1 expression increased Akt activity [4]. In the current study we found that DDAH1 expression decreased the NF1 level. These opposing effects of DDAH1 on NF1 expression and Akt activity are consistent with the GAP function of NF1 and its effect on Ras/Akt pathway. To directly test the effect of NF1 on Akt activity, we knocked down the NF1 level using NF1 sRNAi in HUVEC. Knockdown of NF1 increased pAkt-473 (Figure 8A) and increased cell proliferation (Figure 8B). This result is consistent with a previous study [26]. Furthermore, using DDAH1 knockdown cells, we found that NF1 knockdown restored the decrease in cell proliferation caused by DDAH1 knockdown (Figure 8B).

## Discussion

We previously reported that DDAH1 knockdown inhibited HUVEC proliferation, while overexpression of DDAH1 increased proliferation. In the present study we found that DDAH1 knockdown caused evidence of G1 and G2/M arrest in HUVEC. Control of cell proliferation by extracellular signals can occur at checkpoints in different phases of the cell cycle that are exerted by cyclins, cyclin-dependent kinases (CDKs) and CDK inhibitors (CKI) [27]. Cell cycle progression is triggered by activation of several CDKs; the association of CDKs with cyclins and their inhibitors determines these activations. Cyclin D1 and cyclin E, in association with CDK4 and CDK6, contribute to the G1/S transition, whereas inhibition of the kinase activity of the cyclin/CDK complex is mediated by CKIs including p21cip and p27kip [28]. When cells progress from G2 to M phase, at least two events must occur to activate the mitotic CDC2 kinase: CDC2 must bind with an appropriate B-type cyclin and be dephosphorylated by CDC25 phosphatases. Up-regulation of CKI proteins can inhibit cyclin-CDK complexes and cause cell cycle arrest [29]. In the present study, DDAH1 knockdown decreased cyclin E, cyclin D1, CDC2 and CDC25C expression, which resulted in G1, G2/M arrest and inhibition of cell proliferation. The lack of change of p21cip and p27kip expression implies that these inhibitors did not play a significant role in the cell cycle arrest produced by DDAH1 knockdown.

The effect of DDAH1 on cell cycle progression was also confirmed when we compared MEF from WT and DDAH1 KO mice. We found G2/M arrest in the DDAH1 KO MEF, with decreased cyclin D1 expression, whereas cyclin E, cyclin A, cyclin B, CDK2, CDK4, and P21, P27 were unchanged. These data suggested that DDAH1 may affect cell cycle progression differently on different cell types.

In endothelial cells VEGF is important for cell survival, proliferation, migration and angiogenesis [30]. The VEGF signaling pathway regulates vascular function, at least in part, by controlling NO synthesis. Hasegawa et al [31] found that VEGF mRNA and protein expression were increased in DDAH2-overexpressing endothelial cells, but not in DDAH1-overexpressing cells. In that study, RNAi targeting DDAH2 reduced VEGF production by 44% (they did not study DDAH1 knockdown). In the present study DDAH1 knockdown caused a similar decrease in VEGF protein level, while VEGF was increased in DDAH1 overexpressing cells. We used adenovirus –DDAH1 in the present study while Hasegawa et al used plasmid –DDAH1. It is possible that this technical difference is responsible for the discrepancy between this study and the previous one. Further, we found that the increased VEGF expression produced by DDAH1 overexpression was blocked by Ad-dnRas infection or manumycin A treatment, both of which also abolished the Akt activation [4]. This result indicates that VEGF induction is a down stream effect of Ras/PI3K/Akt activation by DDAH1, which is consistent with a previous study showing that Akt increased VEGF expression by increasing phosphorylation of Sp1 and its binding to the VEGF promoter [32].

DDAH1 is the major enzyme that degrades the endogenous NOS inhibitor ADMA. In DDAH1 transgenic mice, ADMA was decreased while NO production and angiogenesis were increased [7]. Conversely, in the DDAH1 KO mice, ADMA was increased with a consequent decrease in NO production and angiogenesis [2]. NO has an interesting dual role in vascular cell cycle regulation. Thus, NO inhibits vascular smooth muscle cell proliferation in injury models *in vivo* [17] and in cell culture [33], whereas it stimulates endothelial cell DNA synthesis and proliferation via cGMP-dependent transcription [34] and is a central regulator of ischemic angiogenesis [35], [36]. Although the role of NO in cell signaling has been extensively studied, little is known about direct involvement of eNOS/NO in cell cycle regulation. Dai and Faber studied eNOS/NO influences on collateral vessel remodeling by array-profiling collateral vessels from eNOS-KO mice [37]. In remodeling hind limb collaterals isolated 24 hours after femoral artery ligation, they found up-regulation of 44 cell cycle genes in WT mice. In contrast, almost none of these cell cycle genes were up-regulated in the eNOS-KO mice after femoral artery occlusion, in association with impaired proliferation of vascular wall cells. In the DDAH1 global KO mice, both urinary and plasma NOx content were significantly decreased, implying that increased levels of the NOS inhibitor ADMA in the DDAH1 KO mice inhibited NOx generation [2]. It suggested that the decreased angiogenesis in the DDAH1 KO mice we found in this study, at least in part through inhibited NO generation.

DDAH1 has been identified as a NF1-interacting protein that associates with regions of NF1 containing specific PKA phosphorylation sites [22]. Binding of DDAH1 to NF1 increases NF1 phosphorylation by PKA, and increases its association with protein 14-3-3, thereby negatively regulating NF1 GAP function [38] and increasing Ras signaling, events upstream of PI3K, MAPK and Rho. Perturbed up-regulated cell cycle and DNA repair pathways have been found in NF1-haploinsufficient humans and mice [39]. The increase in NF1 seen in the DDAH1 knockdown cells in the present study might be expected to decrease Ras signaling, an effect that could have contributed to the growth arrest observed in the HUVEC.

We previously found increased Ras/Akt activation in DDAH1 overexpressing cells. Conversely, Akt activation was decreased in HUVEC with DDAH1 knockdown as well as in aorta from DDAH1 KO mice [4]. In this study we found that Akt activation was also decreased in DDAH1 KO MEF. Akt has several pathways that can regulate the level of cyclin D1 [16], including enhanced transcription and translation of cyclin D1, and regulation of cyclin D1 protein turnover by inhibition of GSK-3-mediated cyclin D1 phosphorylation. Therefore, the decreased Akt activation in DDAH1 KO or knockdown may account for the down-regulation of cyclin D1, which in turn would lead to cell cycle arrest in the G1 phase. At the G2/M transition, the inhibitory effect of CDC2 phosphorylation (by WEE1Hu) is reversed by dephosphorylation by the CDC25C phosphatase. Akt phosphorylates CDC25C and promotes the translocation of the phosphatase CDC25C from the cytoplasm to the nucleus to activate CDC2. Akt can also phosphorylate and inactivate WEE1Hu to promote cell cycle progression at the G2/M transition [40]. Thus, in addition to contributing to G1 arrest, the decreased Akt activity following DDAH1 knockdown could have contributed to the observed G2/M phase arrest.

Whereas the association between DDAH1/ADMA/NO and angiogenesis is well known, the direct involvement of DDAH1 in cell cycle regulation is largely unknown. Previous studies from our lab and others found that in addition to degrading ADMA, DDAH1 interacts with proteins such as NF1 and Ras [22], [4]. Since both NO and these DDAH1 binding partners can regulate cell cycle genes, the altered cell cycle distribution we found in this study might be the combined consequence of several effects of DDAH1.

In summary, DDAH1 KO impaired both *in vivo* Matrigel angiogenesis and the regeneration of carotid artery endothelium after wire injury. DDAH1 deficiency caused G1 and G2/M arrest in HUVEC that was associated with decreased expression of cyclin D1, cyclin E, CDC2 and CDC25C. We also found that DDAH1 deficiency decreased VEGF and increased NF1 expression. Together the findings indicate that DDAH1 acts through multiple pathways that regulate NO production, the Ras/PI3K/Akt pathway, and VEGF and NF1 expression, the combination of which can alter cell cycle progression, impair endothelial cell proliferation and decrease angiogenesis in DDAH1 KO mice. The DDAH/ADMA pathway has been considered as a potential therapeutic target to treat cardiovascular disease, lung disease, renal disease and cancer [41]. The present study provides further understanding of the mechanism by which DDAH1 can influence endothelial function. The findings provide background information that may be useful in the development of therapeutic strategies to manipulate DDAH1 expression in cardiovascular diseases or tumor angiogenesis.
